# Supplementary material for: Are Probiotics and Prebiotics Safe for Use during Pregnancy and Lactation? A Systematic Review and Meta-Analysis
Source: Nutrients. 2021 Jul 13;13(7):2382. doi: 10.3390/nu13072382 (PMC8308823; doi:10.3390/nu13072382)
Supplement: Supplementary file 1 [file nutrients-13-02382-s001.zip › probiotics AE review supplementary figures final.pdf]

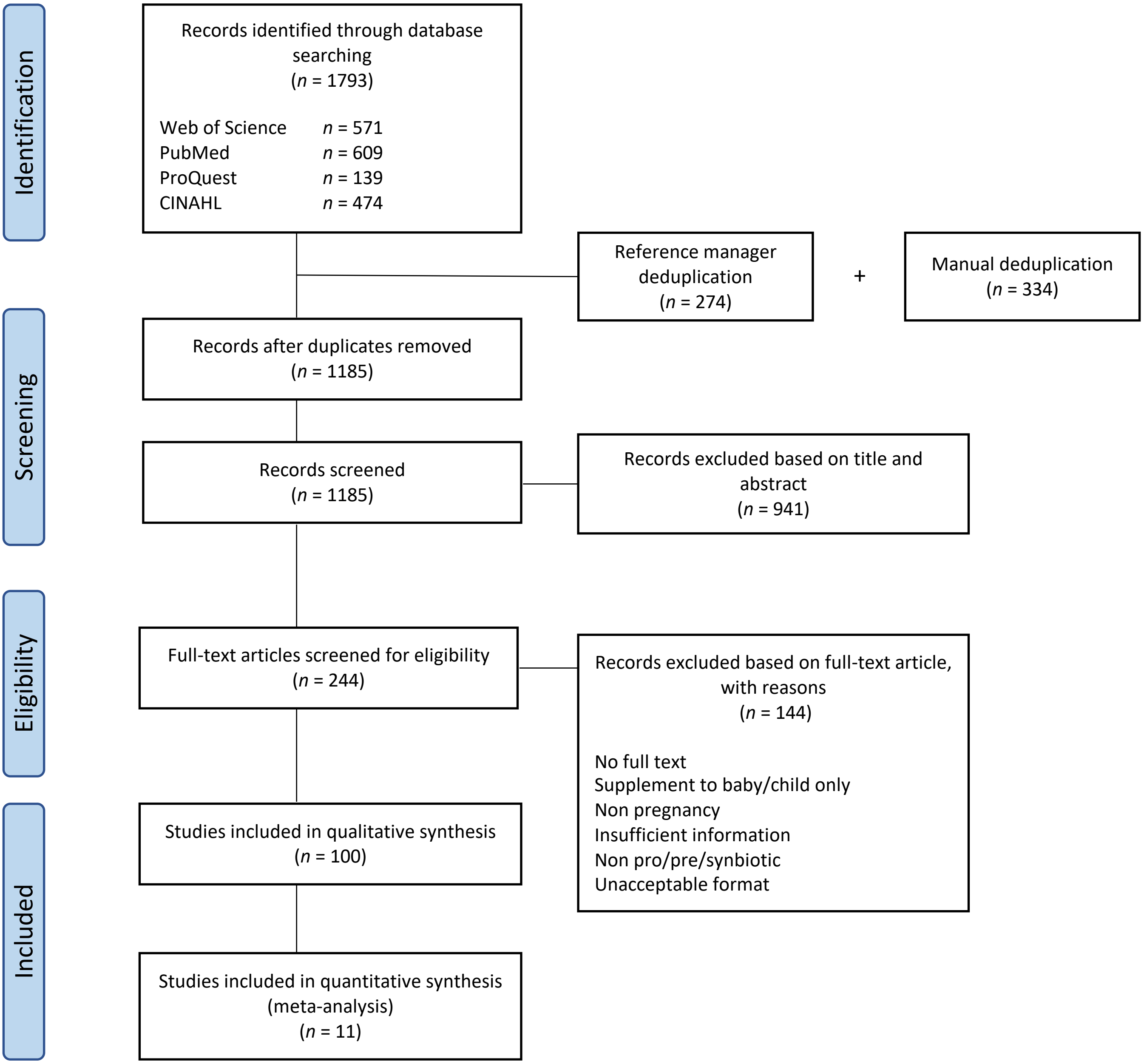


**Supplementary Figure S1.** PRISMA flow diagram for article selection and review.

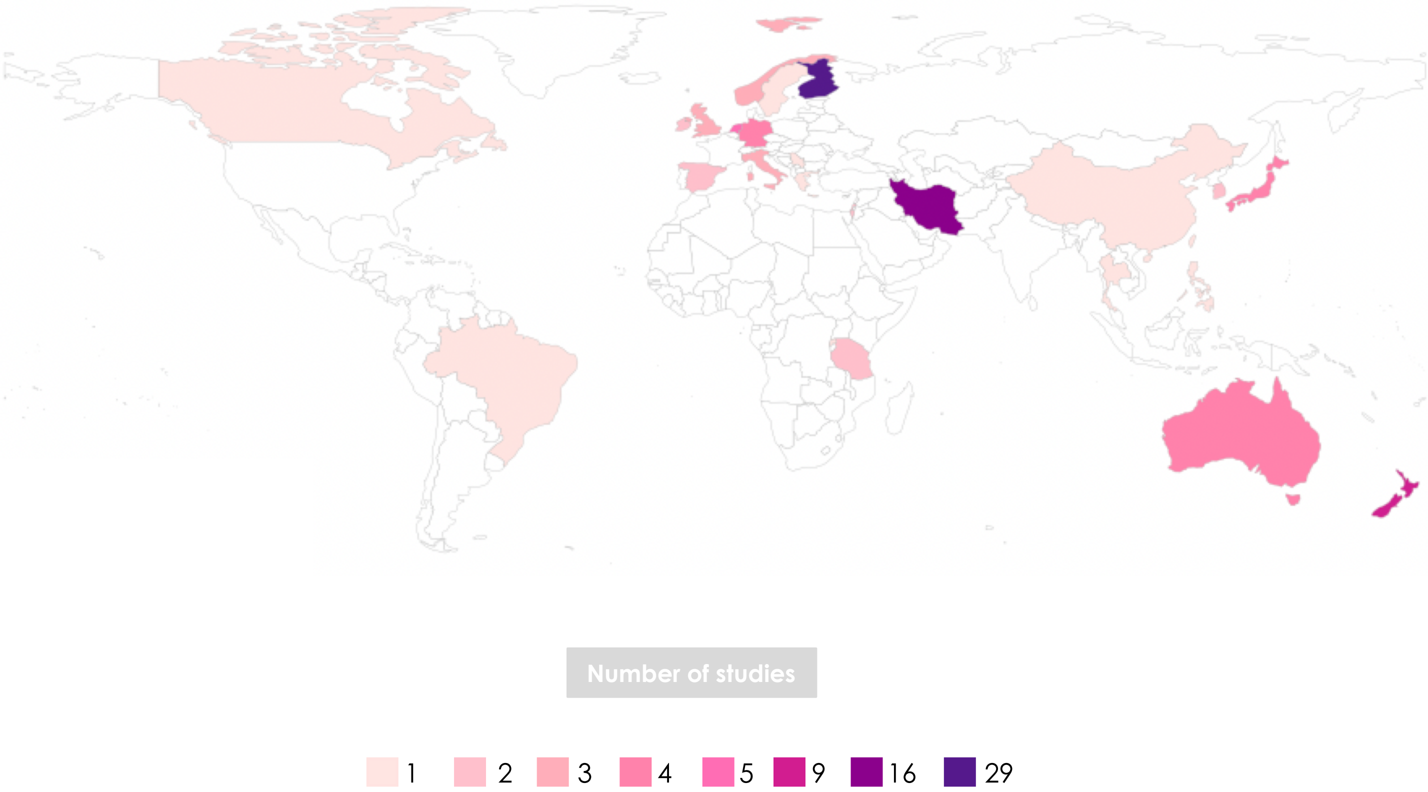


**Supplementary Figure S2.** World heatmap illustrating geographical location of each study included in the review. Data are shown for 100 studies from 25 countries. Increasing study counts are represented by darker colours. Fifty-four percent of studies were from Europe (*n*=54), 28% from Asia (*n*=28), 13% from Oceania (*n*=13), and the remaining 6% from North America, South America and Africa (*n*=6). Finland had the highest representation in studies under review, representing 29% of all studies (*n*=29).

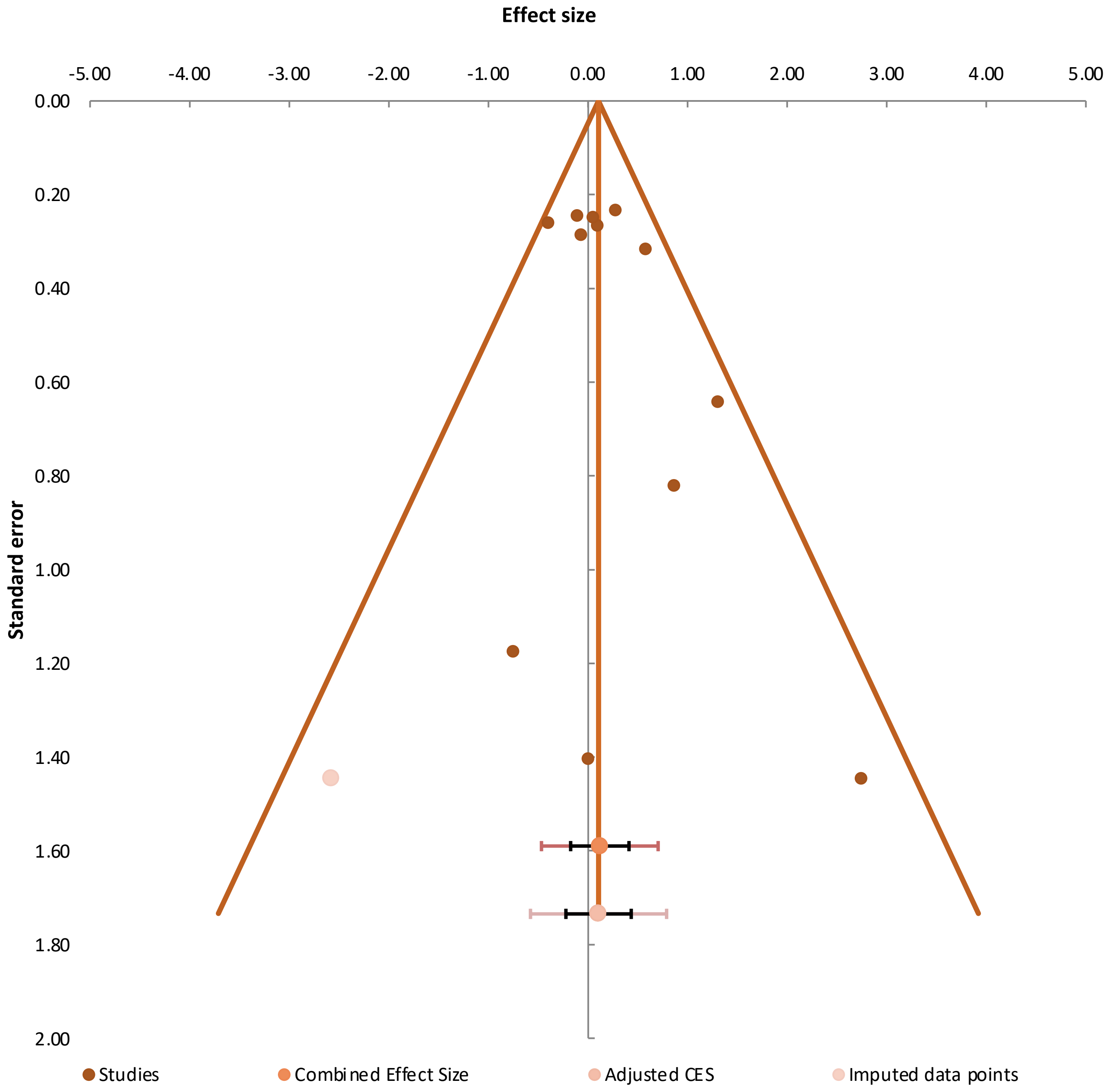


**Supplementary Figure S3.** Funnel plot of log risk ratio values against standard error for each study with reported adverse effects (*n* = 11 studies). One study (Rautava et al.), with two intervention groups and one control group, was treated as two individual studies (thus two dots are represented on the funnel plot) to account for adverse effects that were reported in both intervention groups. The vertical line represents the summary effect size estimates (log risk ratio), and the slanted lines are pseudo 95% CIs. The combined effect size (CSE) is the log risk ratio of all studies combined. The adjusted combined effect size and accompanying confidence and prediction intervals in this plot represent the results of a trim-and-fill procedure. These adjusted statistics are based upon the set of initially included studies expanded with the imputed data points. Funnel plot shows little asymmetry.
