# Supplementary material for: Are Probiotics and Prebiotics Safe for Use during Pregnancy and Lactation? A Systematic Review and Meta-Analysis
Source: Nutrients. 2021 Jul 13;13(7):2382. doi: 10.3390/nu13072382 (PMC8308823; doi:10.3390/nu13072382)
Supplement: Supplementary file 1 [file nutrients-13-02382-s001.zip › probiotics AE review supplementary tables final.pdf]

Supplementary Table S1. Preferred Reporting Items for Systematic Review and Meta-Analysis (PRISMA). This checklist was used as a guideline to determine eligibility criteria, information sources and search strategy, data collection and analysis, and risk of bias assessments and data synthesis.

Section/topic	#	Checklist item	Reported on page #
**TITLE**			
Title	1	Identify the report as a systematic review, meta-analysis, or both.	1
**ABSTRACT**			
Structured summary	2	Provide a structured summary including, as applicable: background; objectives; data sources; study eligibility criteria, participants, and interventions; study appraisal and synthesis methods; results; limitations; conclusions and implications of key findings; systematic review registration number.	1
**INTRODUCTION**			
Rationale	3	Describe the rationale for the review in the context of what is already known.	1
Objectives	4	Provide an explicit statement of questions being addressed with reference to participants, interventions, comparisons, outcomes, and study design (PICOS).	2
**METHODS**			
Protocol and registration	5	Indicate if a review protocol exists, if and where it can be accessed (e.g., Web address), and, if available, provide registration information including registration number.	N/A
Eligibility criteria	6	Specify study characteristics (e.g., PICOS, length of follow-up) and report characteristics (e.g., years considered, language, publication status) used as criteria for eligibility, giving rationale.	2 & 3
Information sources	7	Describe all information sources (e.g., databases with dates of coverage, contact with study authors to identify additional studies) in the search and date last searched.	3
Search	8	Present full electronic search strategy for at least one database, including any limits used, such that it could be repeated.	2 & 3
Study selection	9	State the process for selecting studies (i.e., screening, eligibility, included in systematic review, and, if applicable, included in the meta-analysis).	3
Data collection process	10	Describe method of data extraction from reports (e.g., piloted forms, independently, in duplicate) and any processes for obtaining and confirming data from investigators.	3
Data items	11	List and define all variables for which data were sought (e.g., PICOS, funding sources) and any assumptions and simplifications made.	3 & 4
Risk of bias in individual studies	12	Describe methods used for assessing risk of bias of individual studies (including specification of whether this was done at the study or outcome level), and how this information is to be used in any data synthesis.	4
Summary measures	13	State the principal summary measures (e.g., risk ratio, difference in means).	3 & 4
Synthesis of results	14	Describe the methods of handling data and combining results of studies, if done, including measures of consistency (e.g., I^2) for each meta-analysis.	3, 4 & Fig S3

Section/topic	#	Checklist item	Reported on page #
Risk of bias across studies	15	Specify any assessment of risk of bias that may affect the cumulative evidence (e.g., publication bias, selective reporting within studies).	4
Additional analyses	16	Describe methods of additional analyses (e.g., sensitivity or subgroup analyses, meta-regression), if done, indicating which were pre-specified.	N/A
**RESULTS**			
Study selection	17	Give numbers of studies screened, assessed for eligibility, and included in the review, with reasons for exclusions at each stage, ideally with a flow diagram.	5 & Fig S1
Study characteristics	18	For each study, present characteristics for which data were extracted (e.g., study size, PICOS, follow-up period) and provide the citations.	10 & Fig 4 & Table S4
Risk of bias within studies	19	Present data on risk of bias of each study and, if available, any outcome level assessment (see item 12).	6 & Fig 2
Results of individual studies	20	For all outcomes considered (benefits or harms), present, for each study: (a) simple summary data for each intervention group (b) effect estimates and confidence intervals, ideally with a forest plot.	8, 9, 10 & Fig 4
Synthesis of results	21	Present results of each meta-analysis done, including confidence intervals and measures of consistency.	10 & Fig 4
Risk of bias across studies	22	Present results of any assessment of risk of bias across studies (see Item 15).	6 & Fig 2
Additional analysis	23	Give results of additional analyses, if done (e.g., sensitivity or subgroup analyses, meta-regression [see Item 16]).	10 & Fig S3
**DISCUSSION**			
Summary of evidence	24	Summarize the main findings including the strength of evidence for each main outcome; consider their relevance to key groups (e.g., healthcare providers, users, and policy makers).	12, 13, 14
Limitations	25	Discuss limitations at study and outcome level (e.g., risk of bias), and at review-level (e.g., incomplete retrieval of identified research, reporting bias).	13
Conclusions	26	Provide a general interpretation of the results in the context of other evidence, and implications for future research.	14
**FUNDING**			
Funding	27	Describe sources of funding for the systematic review and other support (e.g., supply of data); role of funders for the systematic review.	N/A

Supplementary Table S2. Classification for primary outcome groups.

Primary Outcome Group	Definition
Maternal microbiome and gastrointestinal health	Involving the maternal microbiota at multiple body sites, including vaginal and gut microbiomes, and gastrointestinal symptoms, such as constipation.
Maternal metabolic health	Involving maternal insulin metabolism, plasma glucose, glycemic status, gestational diabetes mellitus, overweight and obesity, anthropometric measurements, and serum lipid and leptin concentrations.
Maternal mental health	Involving maternal mental health, depression and anxiety.
Maternal breastmilk and breast health	Involving infectious mastitis, breastmilk composition, breastmilk microbiota and immunomodulatory effects in lactation.
Infant microbiome and gastrointestinal health	Involving the infant gut microbiome and gastrointestinal symptoms, such as diarrhea.
Infant metabolic health	Involving infant blood pressure and metabolic programming and child blood lipid concentrations.
Infant allergy and immune health	Involving infant eczema and other allergic disease.
Infant growth and behaviour	Involving infant health outcomes (birthweight, disease symptoms and onset), growth and sleep duration.
Pregnancy outcome	Involving pregnancy rate post-*in vitro* fertilization, incidence and prevention of preterm delivery, incidence of pre-eclampsia and premature membrane rupturing.

Supplementary Table S3. Absolute risk difference (ARD) for studies (*n*=11) with reported adverse effects.

Study	Adverse Effect Type	Absolute Risk Difference (95% CI)
Asgharian, 2020	• Stomachache	0 (-4.30, 4.30)
Gille, 2016	• Increased vaginal discharge<br>• Changes in stool consistence	-5.00 (-9.44, -0.55)
Mirghafourvand, 2016	• Nausea<br>• Bloating<br>• Diarrhea	3.30 (-21.8, 28.5)
Boyle, 2011	• Gastrointestinal symptoms, unspecified	8.00 (-1.86, 17.9)
Krauss-Silva, 2011	• Nausea<br>• Vomiting<br>• Diarrhea<br>• Sleepiness<br>• Headache<br>• Pyrosis<br>• Stomachache<br>• Nausea/vomiting and headache<br>• Vomiting and diarrhea<br>• Pruritus<br>• Abdominal pain/fainting<br>• Abdominal ache<br>• Abdominal pain/vomiting<br>• Bloody diarrhea<br>• Tachycardia	1.00 (-3.47, 5.57)
Shadid, 2007	• Constipation and bloating<br>• Diarrhea or reflux	6.60 (-13.0, 26.3)
Pellonpera, 2019	• Gastrointestinal symptoms, unspecified<br>• Headache	-2.10 (-13.9, 9.80)
Mantaring, 2018	• Maternal nausea, vomiting and diarrhea<br>• Infantile serious diarrhea<br>• Infantile diarrhea	-13.8 (-27.9, 0.37)
Rautava, 2012	• Maternal gastrointestinal symptoms, unspecified<br>• Maternal aggravation of eczema<br>• Infantile gastrointestinal symptoms, unspecified	-12.5 (-32.0, 7.08)
	• Maternal gastrointestinal symptoms, unspecified<br>• Infantile gastrointestinal symptoms, unspecified	-1.90 (-21.6, 17.9)
Arroyo, 2010	• Flatulence	-7.3 (-11.8, -2.69)
Laitinen, 2009	• Flatulence, loose stools or constipation	-4.00 (-10.9, 3.07)

A negative ARD signifies increased risk of the adverse effect(s), whereas a positive ARD signifies decreased risk of the adverse effect(s).

Supplementary Table S4. Full citations of studies reporting adverse effects (*n*=11).

Study name	Full citation
Asgharian, 2020	Asgharian, H., Homayouni-Rad, A., Mirghafourvand, M., Mohammad-Alizadeh-Charandabi, S. Effect of probiotic yoghurt on plasma glucose in overweight and obese pregnant women: a randomized controlled clinical trial. *European Journal of Nutrition* **2020**, *59*, 205-215, doi:10.1007/s00394-019-01900-1.
Gille, 2016	Gille, C., Boer, B., Marschal, M., Urschitz, M. S., Heinecke, V., Hund, V., Speidel, S., Tarnow, I., Mylonas, I., Franz, A., Engel, C., Poets, C. F. Effect of probiotics on vaginal health in pregnancy. EFFPRO, a randomized controlled trial. *American Journal of Obstetrics and Gynecology* **2016**, *215*, doi:10.1016/j.ajog.2016.06.021.
Mirghafourvand, 2016	Mirghafourvand, M.R., A. H. Alizadeh, S. M. Fardiazar, Z. Shokri, K. The Effect of Probiotic Yogurt on Constipation in Pregnant Women: A Randomized Controlled Clinical Trial. *Iranian Red Crescent Medical Journal* **2016**, *18*, doi:10.5812/ircmj.39870.
Boyle, 2011	Boyle, R.J., Ismail, I. H., Kivivuori, S., Licciardi, P. V., Robins-Browne, R. M., Mah, L. J., Axelrad, C.. Moore, S., Donath, S., Carlin, J. B., Lahtinen, S. J., Tang, M. L. K. Lactobacillus GG treatment during pregnancy for the prevention of eczema: a randomized controlled trial. *Allergy* **2011**, *66*, 509-516, doi:10.1111/j.1398-9995.2010.02507.x.
Krauss-Silva, 2011	Krauss-Silva, L.M., M. E. L. Alves, M. B. Braga, A. Camacho, K. G. Batista, M. R. R. Almada-Horta, A. Rebello, M. R. Guerra, F. A randomised controlled trial of probiotics for the prevention of spontaneous preterm delivery associated with bacterial vaginosis: preliminary results. *Trials* **2011**, *12*, doi:10.1186/1745-6215-12-239.
Shadid, 2007	Shadid, R.H., M. Knol, J. Theis, W. Beermann, C. Rjosk-Dendorfer, D. Schendel, D. J. Koletzko, B. V. Krauss-Etschmann, S. Effects of galactooligosaccharide and long-chain fructooligosaccharide supplementation during pregnancy on maternal and neonatal microbiota and immunity - a randomized, double-blind, placebo-controlled study. *American Journal of Clinical Nutrition* **2007**, *86*, 1426-1437.
Pellonpera, 2019	Pellonpera, O.M., K. Houttu, N. Vahlberg, T. Koivuniemi, E. Tertti, K. Ronnemaa, T. Laitinen, K. Efficacy of Fish Oil and/or Probiotic Intervention on the Incidence of Gestational Diabetes Mellitus in an At-Risk Group of Overweight and Obese Women: A Randomized, Placebo-Controlled, Double-Blind Clinical Trial. *Diabetes Care* **2019**, *42*, 1009-1017, doi:10.2337/dc18-2591.
Mantaring, 2018	Mantaring, J.B., J. Destura, R. Pecquet, S. Vidal, K. Volger, S. Guinto, V. Effect of maternal supplement beverage with and without probiotics during pregnancy and lactation on maternal and infant health: a randomized controlled trial in the Philippines. *Bmc Pregnancy and Childbirth* **2018**, *18*, doi:10.1186/s12884-018-1828-8.
Rautava, 2012	Rautava, S.K., E. Salminen, S. Isolauri, E. Maternal probiotic supplementation during pregnancy and breast-feeding reduces the risk of eczema in the infant. *Journal of Allergy and Clinical Immunology* **2012**, *130*, 1355-1360, doi:10.1016/j.jaci.2012.09.003.
Arroyo, 2010	Arroyo, R., Martín, V., Maldonado, A., Jiménez, E., Fernández, L., Rodríguez, J. M. Treatment of infectious mastitis during lactation: antibiotics versus oral administration of Lactobacilli isolated from breast milk. *Clin Infect Dis* **2010**, *50*, 1551-1558, doi:10.1086/652763.
Laitinen, 2009	Laitinen, K.P., T. Isolauri, E. Nutr Allergy Mucosal Immunology, In. Probiotics and dietary counselling contribute to glucose regulation during and after pregnancy: a randomised controlled trial. *British Journal of Nutrition* **2009**, *101*, 1679-1687, doi:10.1017/s0007114508111461.
